# Risk factors of acquired T790M mutation in patients with epidermal growth factor receptor-mutated advanced non-small cell lung cancer

**DOI:** 10.7150/jca.37991

**Published:** 2020-02-03

**Authors:** Wen Ouyang, Jing Yu, Zhao Huang, Gang Chen, Yu Liu, Zhengkai Liao, Wei Zeng, Junhong Zhang, Conghua Xie

**Affiliations:** 1 Department of Radiation and Medical Oncology, Zhongnan Hospital, Wuhan University, Wuhan, China; 2 Hubei Key Laboratory of Tumor Biological Behaviors, Zhongnan Hospital, Wuhan University, Wuhan, China; 3 Hubei Clinical Cancer Study Center, Zhongnan Hospital of Wuhan University, Wuhan, China

**Keywords:** non-small cell lung cancer, epidermal growth factor receptor, T790M, tyrosine kinase inhibitor, risk factors

## Abstract

**Background**: It is still controversial to employ osimertinib as the first-line therapy for EGFR-mutated non-small cell lung cancer (NSCLC) patients in practice. The aim of the current study was to explore the risk factors of acquired T790M mutation during EGFR-TKIs therapy, and to identify the potential patients most likely to benefit from first-line osimertinib treatment.

**Methods**: A total of 222 patients with EGFR-mutated (non-T790M) advanced NSCLC were analyzed. The progression-free survival (PFS), overall survival (OS), and cumulative incidence of acquired T790M mutation were calculated with the Kaplan-Meier method. The independent risk factors were investigated with the multivariate analysis.

**Results**: A total of 70 patients acquired T790M mutation and were treated with osimertinib as a second-line treatment. These patients showed a significantly better OS (*P*=0.003) than those without T790M mutation. Multivariate analysis indicated that BMI ≤ 25 (*P*= 0.031), NSE > 17.9 ng/ml (*P*= 0.013) before treatment, and retroperitoneal lymph node (LN) metastasis (*P*= 0.002) were independent risk factors of acquired T790M mutation. At last, the actuarial risks of acquired T790M mutation at 1 year after EGFR-TKI treatment were 6.6% in patients with 0-1 risk factor and 31.5% in patients with 2-3 risk factors.

**Conclusions**: Patients developing acquired T790M mutation during EGFR-TKI treatment had a better OS of osimertinib treatment. Lower BMI, higher NSE before treatment, and retroperitoneal LN metastasis are independent risk factors of acquired T790M mutation. Our study suggested that patients with 2-3 risk factors were highly recommended the first-line osimertinib treatment.

## Introduction

Epidermal growth factor receptor (EGFR) mutations are observed in approximately 10-15% of the Caucasian population 1 and more than 50% of the Asian population 2 with non-squamous non-small cell lung cancer (NSCLC). It is well known that these patients can be treated with first-generation or second-generation EGFR tyrosine kinase inhibitors (TKIs) such as gefitinib, erlotinib, or afatinib 3,4. However, acquired resistance is almost inevitable after a median treatment period of 9-13 months 5. The acquired p.Thr790Met (T790M) point mutation 6-8 accounts for 50-60% resistance to the first- or second-generation EGFR-TKIs regardless of race or ethnic background 8-11. Osimertinib is an oral and irreversible third-generation EGFR-TKI, selective for EGFR mutations including exon 19 deletion, L858R, and T790M mutation 12-14. Based on the results of the AURA clinical program 15-17, osimertinib was approved worldwide to treat metastatic NSCLC patients with T790M mutation who have disease progression during or after EGFR-TKIs therapy.

Recently, the FLAURA study showed an impressive progression free survival (PFS) benefit of osimertinib against gefitinib (18.9 VS.10.2 months) 18, resulting in approval of osimertinib as the first-line treatment for patients with EGFR-mutated advanced NSCLC regardless of T790M mutation status. However, the median overall survival (OS) benefit of osimertinib against gefitinib in FLAURA study does not appear satisfactory (38.6 VS.31.8 months). And some argued that the sequential EGFR-TKIs treatment might not be inferior to the first-line osimertinib treatment in patients who will develop acquired T790M mutation. Moreover, the currently mechanisms of acquired resistance to osimertinib is unclear for clinical treatment. There would be no subsequent EGFR-TKIs treatment except chemotherapy for the patients who progressed after first-line osimertinib treatment. In addition, the first-generation EGFR-TKIs in combination with antiangiogenic agents (bevacizumab, ramucirumab) 19-21 or chemotherapy 22,23 might be a promising treatment for providing a favorable PFS and even OS. For example, in the NEJ009 study, the first-generation EGFR-TKIs combined with chemotherapy could bring a OS of up to 52.2 months. Considering the cost of osimertinib versus the first/second-generation EGFR-TKIs, the first-line osimertinib treatment is still not widely available.

The AURA clinical program 15-17 confirms that osimertinib treatment can prolong the survival of patients with acquired T790M mutated NSCLC 15. In FLAURA study, upfront osimertinib showed better outcomes than the first-line treatment of gefitinib. According to previous studies, only 50-60% EGFR-mutated patients would develop acquired T790M mutation 8-11. Meanwhile, post-study treatment data of FLAURA study reported in ESMO 2019 showed that 95% patients had disease progression in the gefitinib arm, whereas only 30.55% patients could receive osimertinib as the second-line therapy (65% patients received the second-line therapy, of which 47% received osimertinib). The possible reason was some patients who progressed in gefitinib arm might experience PS score decline, or the second biopsies might be not feasible. Therefore, for patients who will develop T790M mutation, these results indirect suggested that the first-line usage of osimertinib would be more beneficial than the sequential EGFR-TKIs treatments. To date, there is no effective method to screen out these patients.

Consequently, this finding prompted us to identify the population subset that is at the highest risk of developing acquired T790M mutation. We established a retrospective single-institutional database including consecutive patients with EGFR-mutated advanced NSCLC between January 2012 and June 2018, to explore the possible risk factors of acquired T790M mutation during first-generation EGFR-TKI treatment. Our study provided effective methods to screen out the patients who are most likely to benefit from the first-line osimertinib treatment.

## Patients and Methods

### Patients

Between January 2012 and June 2018, A total of 229 consecutive patients with EGFR-mutated advanced NSCLC were treated at the Department of Radiation and Medical Oncology, Zhongnan Hospital of Wuhan University. Among the 229 patients, 4 were excluded due to intrinsic T790M mutation, and 3 were excluded due to short EGFR-TKI treatment (≤ 1 month). A total of 222 eligible patients were included in this study. Our inclusion criteria were: (1) NSCLC was confirmed by cytology (17 pts) or histology (205 pts) (World Health Organization, WHO); (2) The patients were clinically diagnosed as stage IIIB (10 pts) or IV (212 pts) (American Joint Committee on Cancer, the 7th Edition); (3) The patients were treatment naive for EGFR-TKI treatment; (4) Initial EGFR mutations were confirmed by real-time fluorescent quantitative PCR (184 pts, ARMS PCR, Amoy Diagnostics Co., Ltd) or Next Generation Sequencing (38 pts, NGS, Amoy Diagnostics Co., Ltd), using histological or cytological specimens from primary or metastatic lesions; (5) The initial EGFR mutations did not contain the concomitant mutation of T790M. All patients received comprehensive assessments within 1 month before treatment, including physical and pathological examination, EGFR mutation test, and TNM stage evaluation. The clinical and treatment characteristics were shown in Table 1.

This retrospective study was approved by the Ethics Committee of Zhongnan Hospital of Wuhan University. Ethics Committee approved oral informed consent, as the data were reviewed and analyzed anonymously. Informed consent was obtained orally from the included patients by telephone.

### Treatment and Follow up

There were 27 patients receiving chemotherapy as the first-line treatment, and the other 195 patients receiving the first-generation EGFR-TKI (gefitinib, erlotinib, or icotinib) treatment initially, with or without radiotherapy. For EGFR-TKIs treatment, was continuously administered, until progression of disease (PD) or intolerable side effects. Treatment beyond disease progression was allowed if the oncologist judged continued clinical benefit. Follow-up examinations were performed every 2 months, including thoracic and abdominal CT scan and brain MRI scan, until death or loss of follow-up. Treatment response was evaluated by Response Evaluation Criteria in Solid Tumors (RECIST) 1.1 including complete response (CR), partial response (PR), stable disease (SD), and PD. PFS was defined as the time from EGFR-TKI treatment to PD (local, regional, or distant progression) or death from any cause. Time to subsequent treatment (TTST) was defined as the time from EGFR-TKI treatment to subsequent treatment or death from any cause. OS was defined as time from the first-generation EGFR-TKI treatment to death from any cause. OS_total_ was defined as time from diagnosis to death from any cause. T790M-developing-free survival (TDFS) was defined as time from the first-generation EGFR-TKI treatment to the occurence of documented post-progression T790M mutation.

### Statistics

All statistical analyses were conducted using Statistical Package for Social Scientists (SPSS Version 22.0, SPSS Inc., Chicago, USA). Descriptive statistics were used for categorical variables (frequency and percentage) and continuous variables (median and range). The cumulative incidence of acquired T790M mutation and survival were calculated by the Kaplan-Meier method with 95% confidence interval (CI). Univariable and multivariable Cox regression analyses were performed to analyze the risk factors of acquired T790M mutation. The multivariable Cox regression analysis simultaneously included factors that had shown associations (*P* < 0.100) in the univariable Cox regression analyses. In addition, patients were stratified into 2 risk groups according to the independent risk factors of multivariable Cox regression analysis. Log-rank survival analysis was performed to examine the difference of survival between these 2 groups. The optimal cut-off values of continuous valuables were calculated by X-tile software 24. All tests were two-sided and *P* < 0.05 were considered statistically significant.

## Results

### Patient characteristics

A total of 229 consecutive patients with EGFR-mutated advanced NSCLC were analyzed. Except 4 patients with intrinsic T790M mutation, and 3 with short EGFR-TKI treatment (< 1 month), 222 eligible patients were enrolled in this retrospective study. Among them, 70 patients acquired T790M mutation during the EGFR-TKI treatment and received third-generation EGFR-TKI therapy, whose T790M mutation were confirmed in plasma (51 pts, ddPCR, KingMed Diagnostics Group Co., Ltd.), cellular (3 pts, ddPCR, KingMed Diagnostics Group Co., Ltd.) or tissue (16 pts, NGS, Genecast Biotechnology Co., Ltd) specimens. All of the 222 patients were analyzed for the risk factors of acquired T790M mutation by univariable and multivariable Cox regression analyses.

### Acquired T790M mutation indicates better outcomes

The median duration of follow-up was 22.8 months (95% CI: 19.3-26.2 months). The median OS of the 222 patients was 37.5 months (95% CI: 26.9-48.1 months). The OS rates of 1-year, 2-year, and 3-year were 88.3%, 64.2%, and 53.4% respectively. The median OS_total_ of the 222 patients was also 37.5 months (95% CI: 27.7-47.3 months). The OS_total_ rates of 1-year, 2-year, and 3-year were 89.0%, 65.4%, and 55.1% respectively.

**Figure 1:**
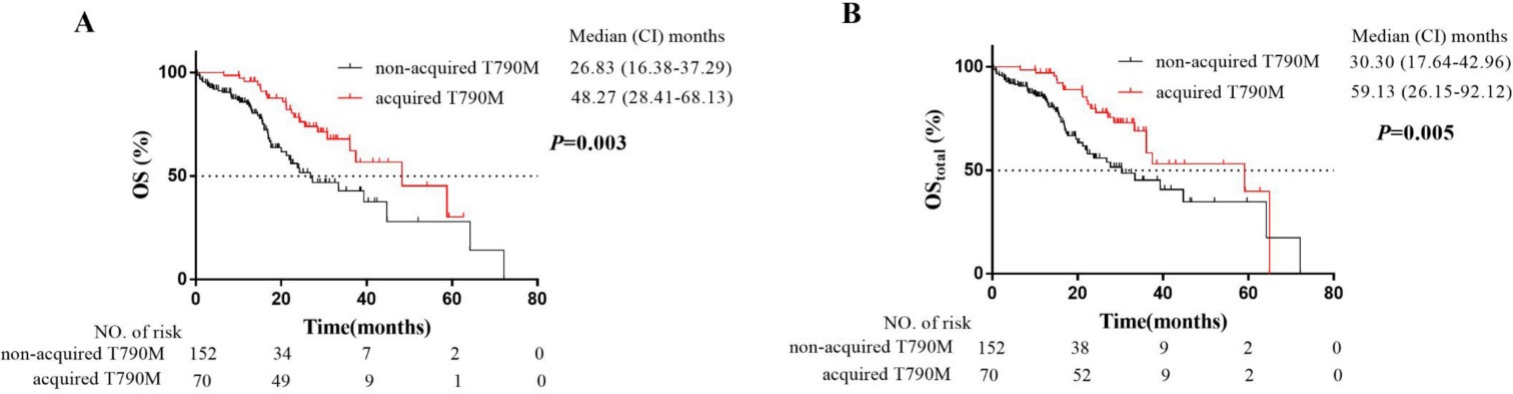
Kaplan-Meier plot of OS **(A)** and OS_total_ **(B)** in EGFR-mutated advanced NSCLC patients with or without acquired T790M mutation. OS, overall survival from the first-generation EGFR-TKI treatment; OS_total_, overall survival from initial treatment (the first-generation EGFR-TKI treatment or chemotherapy): CI, confidence interval.

To evaluate the effect of acquired T790M mutation on OS, Log-rank comparisons of OS were performed based on T790M mutation status. Patients with acquired T790M mutation had better outcomes (median OS: 48.3 months, median OS_total_: 59.1 months) than patients without T790M mutation (median OS: 26.8 months, median O_Stotal_: 30.3 months). The survival curves were shown in Fig.1. Our median OS was longer than those of previous clinical trials of EGFR-TKI treatment for EGFR-mutated advanced NSCLC patients 25, which was largely attributed to the usage of osimertinib.

### Acquired T790M mutation had no impact on PFS

**Figure 2:**
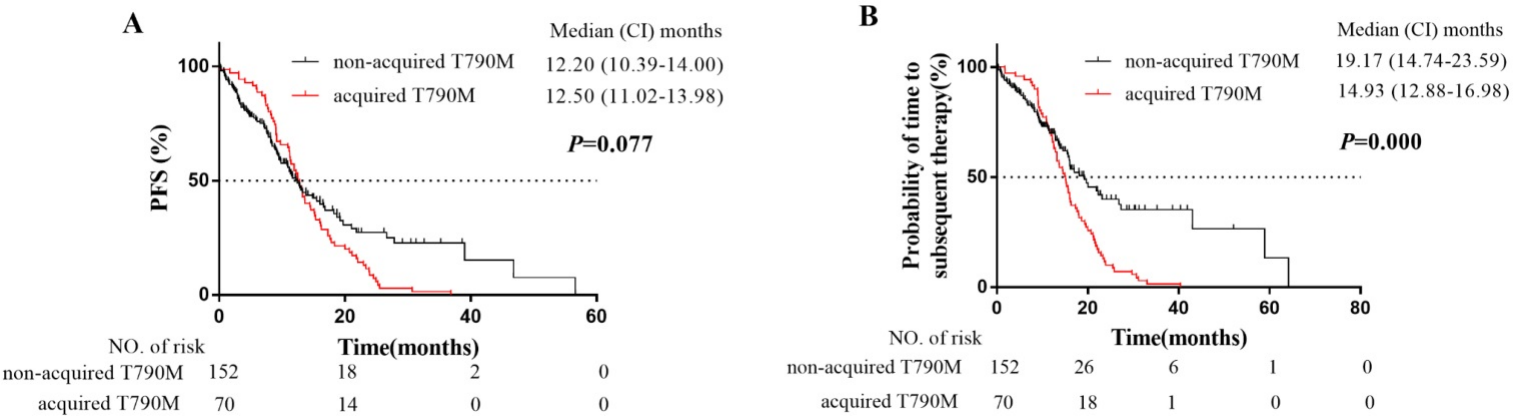
Kaplan-Meier plot of PFS **(A)** and TTST **(B)** in EGFR-mutated advanced NSCLC patients with or without acquired T790M mutation. PFS, progression-free survival from the EGFR-TKI treatment to PD or death; TTST, time to subsequent treatment from the EGFR-TKI treatment to subsequent treatment or death; CI, confidence interval.

The median PFS of the 222 patients was 12.4 months (95% CI: 11.3-13.6 months). The PFS rates of 1-year, 2-year, and 3-year were 51.7%, 17.1%, and 10.3% respectively (Fig. 2A). A total of 159 patients (71.6%) had PD for the first time during follow-up period. Among them, the number of patients with local progression, slow progression, and rapid progression was 73 (45.9%), 39 (24.5%), and 47 (29.6%) respectively. In addition, the median PFS of patients with acquired T790M mutation was 12.5 months (95% CI: 11.0-14.0 months), and the median PFS of patients without T790M mutation was 12.2 months (95% CI: 10.4-14.0 months) (Fig. 2A). The acquired T790M mutation did not significantly influence on the PFS of the first-generation EGFR-TKIs therapy (*P* = 0.077).

Furthermore, EGFR-TKIs treatment beyond disease progression was allowed if the oncologist judged continued clinical benefit. Therefore, 82 patients (82/159, 51.6%) with local progression or slow progression received continuous the first-generation EGFR-TKIs treatment with or without radiotherapy. Whereas, 26 patients (16.4%) switched to chemotherapy, 35 patients (22%) switched to the third-generation EGFR-TKIs, and 16 patients (10.1%) received supportive care. The median TTST of the 222 patients was 16.0 months (95% CI: 14.6-17.4 months). While the median TTST of the patients with acquired T790M mutation was 14.9 months (95% CI: 12.9-17.0 months), and the median TTST of patients without T790M mutation was 19.2 months (95% CI: 14.7-23.6 months) (Fig. 2B). The duration of first-generation EGFR-TKI treatment for the patients without T790M mutation was significantly longer than that for the patients with acquired T790M mutation (*P* = 0.000, Fig. 2B). It was largely attributed to the intervention of radiotherapy for oligometastatic (9 pts developed acquired T790M mutation, and 27 pts didn't develop acquired T790M mutation) and oligoprogressive (14 pts developed acquired T790M mutation, and 29 pts didn't develop acquired T790M mutation) metastases in patients during the EGFR-TKIs treatments.

### The incidence of acquired T790M mutation and post-treatment

**Figure 3:**
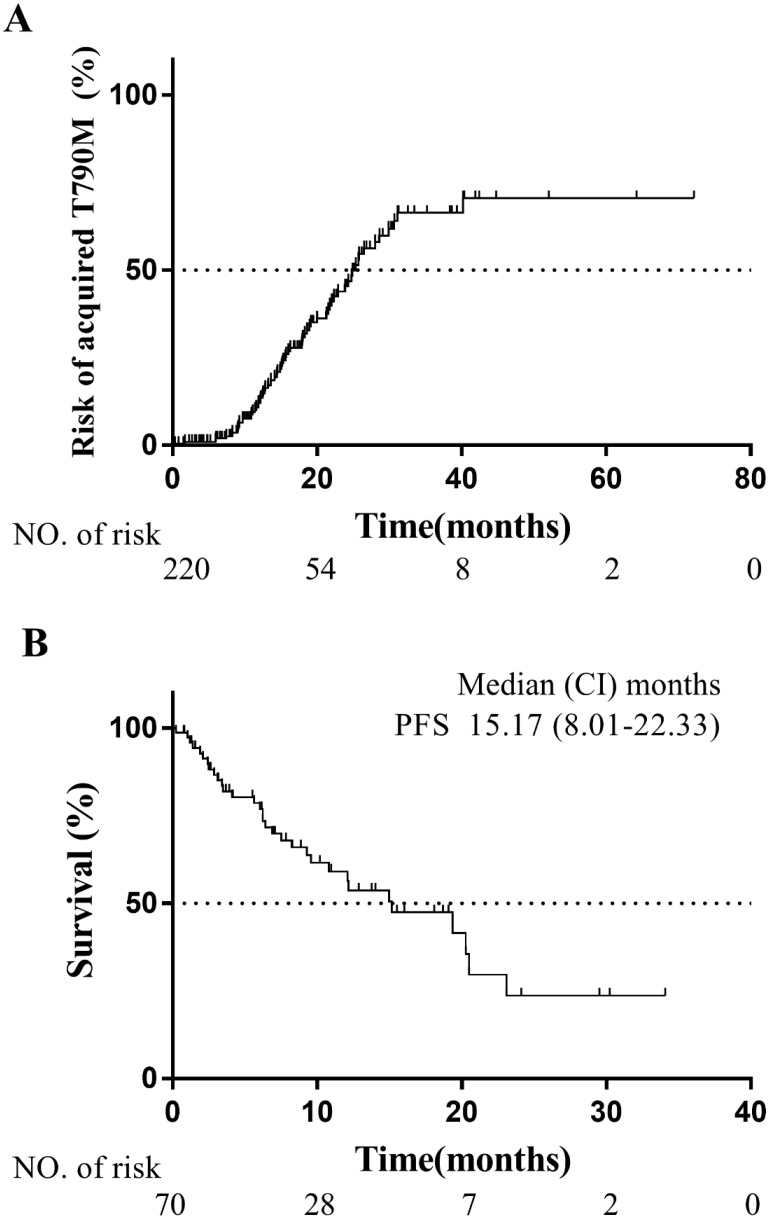
** (A)** Kaplan-Meier plot of acquired T790M mutation risks in patients with EGFR-mutated advanced NSCLC. **(B)** Kaplan-Meier plot of PFS in T790M-mutated patients treated with osimertinib. PFS, progression-free survival from osimertinib treatment to PD or death; CI, confidence interval.

Between January 2012 and June 2018, 159 patients had PD for the first time. All of them firstly received the detection of T790M mutation by droplet digital PCR (ddPCR, KingMed Diagnostics Group Co., Ltd.) using plasma specimens. Among them, 51 patients were confirmed T790M mutation. Then 46 patients with plasma T790M mutation negative received tissue rebiopsy for T790M mutation detection by NGS (16 pts confirmed T790M mutation, Genecast Biotechnology Co., Ltd), and the malignant pleural effusion of 9 patients were detected by ddPCR (3 pts confirmed T790M mutation, KingMed Diagnostics Group Co., Ltd.). A total of 70 patients were confirmed to develop acquired T790M mutation during the EGFR-TKI treatment. The positive rate of acquired T790M mutation was 44.0%, which was consistent with previous studies 8-11. The median TDFS was 24.9 months (95% CI: 21.9-27.9 months), and the risk of developing acquired T790M mutation at 1-year, 2-year, and 3-year was 12.1%, 45.3%, and 66.3% respectively (Fig. 3A).

All of the 70 patients with acquired T790M mutation received subsequent therapy of the third-generation EGFR-TKI (osimertinib). The median duration of osimertinib treatment was 15.2 months (95% CI: 8.0-22.3 months) (Fig. 3B). On account of the intervention of radiotherapy for oligoprogressive metastases during osimertinib treatment, our results of the median duration of osimertinib treatment were longer than PFS of the AURA3 study for advanced NSCLC patients with T790M mutation in osimertinib subsequent-line therapy 15.

### Risk factors of developing acquired T790M mutation

In univariate analyses, the lower BMI of patients, higher CEA and NSE level before treatment, liver metastasis, bone metastasis, and retroperitoneal lymph node (LN) metastasis were associated with increased risks of acquired T790M mutation. No significant association was found between acquired T790M mutation and other factors, such as gender, age, KPS score, smoking status, CA125 level before treatment, first-line treatment regimen, intervention of radiotherapy, type of EGFR mutations at initial diagnosis, and other location of metastatic sites (Table 2).

**Figure 4:**
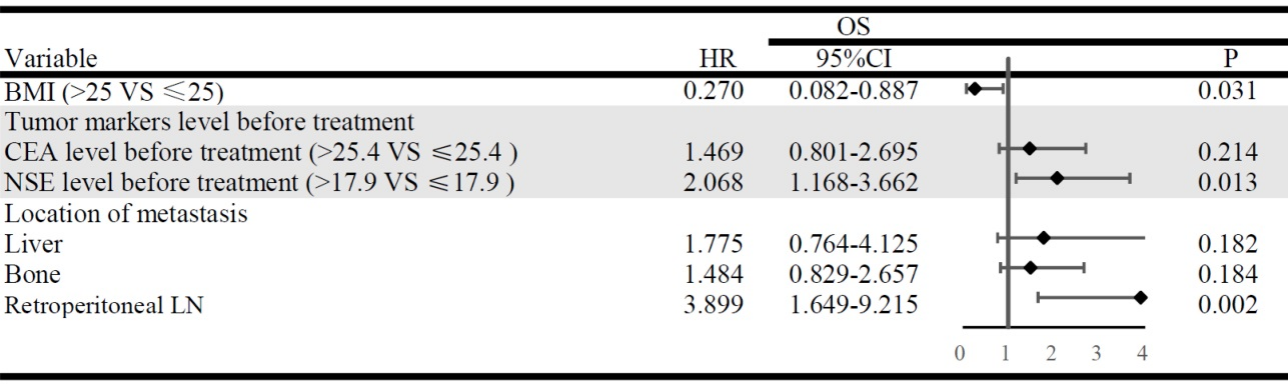
Multivariate analysis and forest plots indicate the independent risk factors of acquired T790M mutation. HR, hazard ratio; CI, confidence interval.

The multivariable analysis simultaneously included factors that had shown associations (*P* < 0.100) in the univariable analyses. The results indicated that BMI ≤ 25 (*P* = 0.031), NSE > 17.9 ng/ml before treatment (*P* = 0.013) and retroperitoneal LN metastasis (*P* = 0.002) were the independent risk factors of acquired T790M mutation. Whereas, liver metastasis, bone metastasis, and CEA levels before treatment were not significantly associated with acquired T790M mutation (Fig. 4).

**Figure 5:**
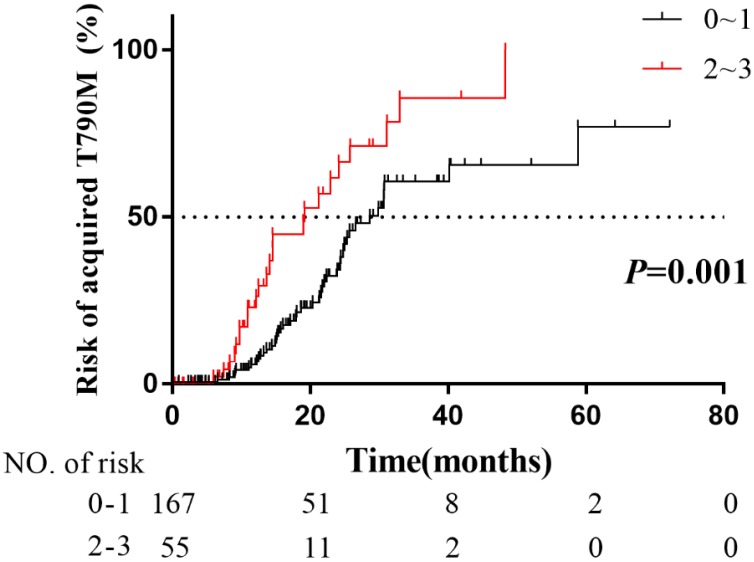
Comparison of the actuarial risk of acquired T790M mutation among patients with different numbers of risk factors.

Furthermore, patients were stratified into 2 risk subgroups: patients with 0~1 (n = 167) risk factor as the low-risk group, and patients with 2~3 (n = 55) risk factors as the high-risk group. The actuarial risk of developing acquired T790M mutation at 1 year were 6.6% in the low-risk group and 31.5% in the high-risk group (*P* = 0.001, Fig. 5). Obviously, patients with 2~3 risk factors had a higher risk of developing acquired T790M mutation. Our studies suggested that the patients with 2~3 risk factors were candidates for the third-generation EGFR-TKIs as the first-line therapy.

## Discussion

Before the approval of EGFR-TKIs, the median OS of advanced NSCLC patient was no more than 1 year 26. Whereas the discovery and advances of EGFR-TKIs revolutionarily improved prognosis of EGFR-mutated advanced NSCLC patients. The clinical trials of the first- or second-generation EGFR-TKIs showed a median OS of 19.3-33.2 months 25,27. Similarly, osimertinib, as one of the third-generation EGFR-TKIs that selectively inhibit T790M mutation 14, significantly improves the survival of patients with T790M mutation 15-17. Our study firstly evaluated the effect of acquired T790M mutation on OS. The patients without T790M mutation had a significantly higher risk of OS compared with the patients with acquired T790M mutation. In detail, our median OS of the 222 patients with EGFR-mutated advanced NSCLC was 37.5 months (95% CI: 26.9-48.1 months). Patients with acquired T790M mutation showed a median OS of 48.3 months (95% CI:28.4-68.1 months) (Fig.1). The better OS in our study than the first-generation EGFR-TKIs clinical trials 25 was attributed to the usage of third-generation EGFR-TKIs for patients with acquired T790M mutation. Therefore, our results also confirmed that the OS of patients with acquired T790M mutation was prolonged by the usage of osimertinib.

Moreover, the median PFS of the 222 patients was 12.4 months, and there was no difference in PFS of the first-generation EGFR-TKIs treatment between patients with acquired T790M mutation group and without T790M mutation group (*P* = 0.077). Our result of median PFS is consistent with the results of the first-generation EGFR-TKIs clinical trials 25. Whereas the median TTST for patients without T790M mutation was 19.2 months, while 14.9 months (*P* = 0.000, Fig.2B) for patients with acquired T790M mutation. It was largely attributed to the intervention of radiotherapy for oligometastatic and oligoprogressive metastases in patients during the EGFR-TKIs treatments, especially for patients without T790M mutation. Our results also confirmed the important role of radiotherapy in the management of EGFR-mutated advanced NSCLC patients.

Between January 2012 and June 2018, 159 patients had PD for the first time and were detected for T790M mutation. A total of 70 patients were documented to acquire T790M mutation. The positive rate of T790M mutation was 44.0%, which was consistent with previous reports 8-11. All of 70 patients with acquired T790M mutation received subsequent osimertinib treatment. The median duration of osimertinib treatment was 15.2 months (95% CI: 8.0-22.3 months) (Fig.3B). The subgroup analysis of the AURA3 study in Japanese patients showed a median PFS of 12.5 months 15. Considering the intervention of radiotherapy for oligoprogressive metastases, our results of the median duration of osimertinib treatment was still consistent.

The FLAURA study shows PFS benefit in the third-generation EGFR-TKI osimertinib than the first-generation EGFR-TKI gefitinib for the first line treatment 18. But considering the cost-effectiveness of osimertinib, the first-line osimertinib treatment for EGFR-mutated advanced NSCLC patients is still not widely available. In our study, there were 159 patients who had PD for the first time during follow-up period, and 53 patients just confirmed plasma T790M mutation negative. Among them, 16 patients had poor performance status, and 17 patients refused the second biopsy, and the lesions of 20 patients are not suitable for rebiopsy. Therefore, on account of some patients who progressed after first/second-generation EGFR-TKIs treatment might have poor performance status, or the second biopsies might be not feasible. At least for patients who would develop acquired T790M mutation, it was strongly recommended the osimertinib first-line treatment rather than the sequential EGFR-TKIs treatments. In order to identify the population subset with higher risk of developing acquired T790M mutation, we performed multivariate Cox regression analysis. Our results indicated that BMI ≤ 25, NSE > 17.9ng/ml before treatment and retroperitoneal LN metastasis were the independent risk factors of developing acquired T790M mutation. To date, there have been no study to explain the underlying mechanisms of the correlation between these risk factors and acquired T790M mutation. The mechanisms are still to be investigated. Furthermore, patients were divided into 2 risk subgroups: patients with 0~1 (n=167) risk factors of low-risk group, and patients with 2~3 (n=55) risk factors of high-risk group. The actuarial risk of developing acquired T790M mutation at 1 year were 6.6% in the low-risk group and 31.5% in the high-risk group. Our study suggested that the patients with 2~3 risk factors were potential candidates for the third-generation EGFR-TKIs as the first-line therapy.

At last, our findings confirmed that the third-generation EGFR-TKI improved the survival of patients with acquired T790M mutation. Lower BMI (≤ 25), higher NSE (> 17.9 ng/ml) before treatment and retroperitoneal LN metastasis were the independent risk factors of acquired T790M mutation. Certainly, there are several limitations in our study, this was a retrospective study in a single institution, which inevitably resulted in a selection bias. The feasibility of selectively using osimertinib as first-line treatment in higher-risk patients should be further confirmed in the future, and the mechanisms of the correlation between these risk factors and acquired T790M mutation is to be explored. However, we firstly presented the view that using osimertinib as first-line treatment in higher-risk patients, which is the theoretical innovation part of this paper. As well as IPASS study, the better biomarkers will be found to predict for acquired T790M mutation by the exploration of risk factors.

## Figures and Tables

**Table 1:** Clinical characteristics of patients

Characteristic	NO.	%
**NO. of all patients with EGFR mutations**	226	
**NO. of patients with intrinsic T790M mutation**	4	1.8
**NO. of patients developing T790M mutation**	70	31.0
**Age, years**	222	
Median(Range)	57(30-93)	
**Gender**	222	
Male	97	43.7
Female	125	56.3
**KPS score**	222	
≥80	199	89.6
<80	23	10.4
**Histology**	222	
Adenocarcinoma	214	96.4
Non-adenocarcinoma carcinoma	8	3.6
**BMI**	219	
≤25	184	84.0
>25	35	16.0
Median(Range)	21.91(13.67-30.82)	
**Smoking status**	222	
Yes	62	27.9
No	160	72.1
**CEA (ng/ml)**	196	
>25.4	98	50.0
≤25.4	98	50.0
Median(Range)	27.23 (0.61-8048.83)	
**CA125 (ng/ml)**	176	
Median(Range)	48.08 (4.76-5304.00)	
**NSE(ng/ml)**	176	
>17.9	62	35.2
≤17.9	114	64.8
Median(Range)	14.61 (4.42-70.87)	
**First-line treatment regimen**	222	
EGFR-TKI treatment	195	87.8
Chemotherapy	27	12.2
**The intervention of radiotherapy or not**	222	
NO. of radiotherapy for oligometastatic metastases	36	16.2
NO. of radiotherapy for oligoprogressive metastases	43	19.4
**Type of EGFR mutations**	222	
Exon 21 point mutation	94	42.3
Exon 19 deletion mutation	119	52.7
Other	11	5.0
**NO. of metastasis**	222	
0	10	4.5
1	103	46.4
2	74	33.3
3 or more	35	15.8
**Location of metastatic sites**	222	
Brain	71	32.0
Pleural effusion	18	8.1
Liver	25	11.3
Adrenal	28	12.6
Bone	116	52.3
Lung	123	55.4
Retroperitoneal LN	14	6.3
Other	6	2.7
**Type of EGFR-TKIs**	222	
Gefitinib	178	80.2
Erlotinib	15	6.8
Icotinib	29	13.1
**Type of post-progression confirmed gene testing**	159	
Tissue	46	28.9
Cellular	9	5.7
Plasma	104	65.4

**Table 2:** Univariate analyses for the risk factors of developing T790M mutation

Factors	HR	95%CI	*P*
**Gender: female VS male**	1.449	0.894-2.348	0.132
**Age, years**	0.994	0.971-1.017	0.587
**KPS score: <80 VS ≥80**	1.422	0.612-3.304	0.414
**BMI**	0.932	0.877-0.990	0.023
>25 VS ≤25	0.371	0.169-0.815	0.014
**Smoking: yes VS no**	0.873	0.515-1.480	0.614
**Tumor markers level before treatment**			
CEA (ng/ml)	1.000	1.000-1.000	0.011
>25.4VS ≤25.4	2.006	1.190-3.381	0.009
CA125 (ng/ml)	1.000	1.000-1.001	0.107
NSE (ng/ml)	1.020	0.999-1.043	0.057
>17.9 VS ≤17.9	2.135	1.221-3.731	0.008
**First-line treatment regimen**			
Chemotherapy VS EGFR-TKI	1.083	0.569-2.064	0.808
**The intervention of radiotherapy or not (Yes VS No)**			
Radiotherapy for oligometastatic metastases	0.954	0.473-1.923	0.894
Radiotherapy for oligoprogressive metastases	0.415	0.151-1.140	0.088
**Type of EGFR mutations**			0.436
19-del VS L858R	1.326	0.806-2.184	0.267
Other VS L858R	1.673	0.576-4.857	0.344
**NO. of metastasis**			0.298
≤1 VS 3 or more	0.651	0.299-1.417	0.279
2 VS 3 or more	0.931	0.420-2.062	0.860
**Location of metastasis**			
Brain	0.909	0.531-1.557	0.728
Pleural effusion	0.788	0.286-2.170	0.645
Liver	2.016	1.025-3.964	0.042
Adrenal	0.506	0.203-1.260	0.143
Bone	1.516	0.941-2.442	0.088
Lung	1.084	0.675-1.743	0.738
Retroperitoneal LN	3.698	1.809-7.560	0.000
Other	0.047	0.000-17.343	0.310
